# The Influence of 2-Methacryloyloxyethyl Phosphorylcholine Polymer Materials on Orthodontic Friction and Attachment of Oral Bacteria

**DOI:** 10.3390/ma15165770

**Published:** 2022-08-21

**Authors:** Ryo Kunimatsu, Yuji Tsuka, Kengo Nakajima, Keisuke Sumi, Yuki Yoshimi, Isamu Kado, Ayako Inada, Yoshihiro Kiritoshi, Kotaro Tanimoto

**Affiliations:** 1 Department of Orthodontics and Craniofacial Development Biology, Graduate School of Biomedical & Health Sciences, Hiroshima University, Hiroshima 734-8553, Japan; 2 Intelligent Surfaces, Inc., Kashiwa 277-0882, Japan

**Keywords:** 2-methacryloyloxyethyl phosphorylcholine, oral bacteria, friction

## Abstract

There is no clinical evidence of the usage of 2-methacryloyloxyethyl phosphorylcholine (MPC) polymers in dental practice. We performed in vitro studies to determine whether the application of an MPC coating to stainless steel orthodontic wires confers low-friction and antimicrobial properties to these wires. The friction test on MPC-coated wires was performed using a precision universal/tensile tester. MPC polymer was coated on a 50 × 50 mm stainless steel plate, and samples were assessed using an antimicrobial activity test. To verify the effect of MPC polymer-treated wires on experimental tooth movement models in vitro, examinations were performed on typodonts to determine the improvement in tooth movement efficiency. The polymer treatment wire groups demonstrated significantly enhanced tooth movement compared with the untreated wire groups, at both 50 g and 100 g traction forces. The results indicated that MPC coating inhibited the attachment of oral bacteria, such as *Streptococcus mutans*, on a stainless steel plate. Additionally, the coating seemed to improve the efficiency of tooth movement by reducing the occurrence of friction. The application of an MPC coating onto stainless steel wires, which are used as orthodontic materials, may reduce static friction and bacterial adherence to the oral cavity and improve tooth movement.

## 1. Introduction

The goal of orthodontic treatment is to induce normal growth and development of the maxillofacial and oral tissues, thereby ensuring good maxillofacial configuration and normal oral function [1]. Therefore, the development of new strategies and materials to improve the efficiency and effectiveness of orthodontic treatment and appliances is necessary.

During mechanical orthodontic treatment performed using multi-bracket appliance systems, friction in the bracket–archwire interfaces may interfere with the action of forces required for specific movements [2]. Multiple studies have demonstrated that approximately 15–60% of the force used to move the teeth dissipates as friction [3,4]. This phenomenon could delay the biological responses to orthodontic movement [5]. Considering these limitations, improving orthodontic teeth movement with orthodontic appliances would potentially enhance treatment efficiency and reduce friction between the wire and bracket appliance. However, despite multiple advances in orthodontic techniques, the occurrence of demineralized white-spot lesions (WSLs) during treatment remains a serious side effect, particularly when using fixed appliances [6]. Therefore, improved preventive measures and antimicrobial materials are urgently required to prevent biofilm-related complications of orthodontic treatment from overshadowing its functional and aesthetic advantages [7].

In recent years, the increasing number of adult patients seeking orthodontic treatment has led orthodontists to face more patients with periodontal disease [8]. Orthodontic treatment planning requires consideration of functional problems resulting from alveolar bone resorption and inflammatory periodontal disease. A recent study has suggested that orthodontic tooth movement can enhance bacteria-induced periodontal inflammation and destruction and that inflammatory cytokines such as interleukin (IL)-6 may play a pivotal role in this process [9]. Moreover, previous studies on tooth movement in response to periodontal lesions using finite element methods (FEMs) have shown that periodontally reduced tooth movement into the alveolar bone is associated with increased stress and load on the periodontal tissues [10,11]. Studies have also demonstrated that smaller mechanics must be chosen to reduce tissue loading compared to that used for normal alveolar bone [10,11]. Therefore, for patients with periodontal disease, it is necessary to develop strategies for orthodontic treatment and orthodontic materials to control periodontitis induced by bacteria and to reduce excessive force and friction.

Since microbial adherence is primarily essential in infection and biofilm formation, the inhibition of microbial adherence is an effective strategy for preventing infectious diseases and biofilm formation [12]. One of the initial steps in the process of dental plaque formation is the adherence of early colonizers primarily composed of gram-positive species, such as *Streptococcus mutans* (*S. mutans*) to the exposed salivary pellicle covering oral tissue surfaces [12]. Co-aggregation, which is characterized by the attachment of secondary colonizers to primary bacteria already anchored to the teeth or tissues, is also important for the accretion of dental plaque [13]. Therefore, inhibition of bacterial adherence and co-aggregation are effective methods of suppressing dental plaque formation and maturation [12]. Furthermore, plaque deposition facilitates bacterial attachment, causing a decrease in pH due to bacterial metabolism [14]. Acidic bacteria in biofilms can reduce the pH of local plaques to 4.5 or 4 by metabolizing carbohydrates [15]. When the pH around the teeth drops below 5.5, decalcification of the teeth becomes remarkable. Further, dissolving the minerals of the whole enamel can damage the teeth, resulting in initial caries and WSL. Education on oral hygiene and fluoride use is recommended to prevent the occurrence of WSL; however, these mitigation measures depend on patient compliance and may not be effective in children and adolescents [16]. Therefore, initial bacterial attachment is a crucial step in WSL and caries formation, and initial salivary protein coating is a prerequisite for bacterial attachment [16]. Based on the above background, orthodontic materials that can inhibit protein adsorption may reduce bacterial adhesion, leading to the prevention or minimization of WSL.

In this regard, 2-methacryloyloxyethyl phosphorylcholine (MPC) polymer coatings, which are coatings of phospholipid polymers with antibacterial, hydrophilic, and low-friction properties, have been developed in the medical field for applications in artificial blood vessels and prostheses [17,18,19]. As the mouth harbors numerous bacterial species depending on the different surface properties, such antimicrobial coatings may help improve the oral environment during orthodontic treatment [20,21,22,23,24]. Based on the aforementioned points, we hypothesized that the coating of MPCs on the stainless steel materials used in orthodontic treatment could prevent oral bacterial adherence to wires, attenuate the friction coefficient, and achieve experimental tooth movement. To establish this hypothesis, we performed an in vitro assessment to determine whether application of an MPC coating to stainless steel wires used in orthodontic materials would enhance the frictional and antimicrobial properties of the materials.

## 2. Materials and Methods

### 2.1. Preparation of MPC Polymer-Coated Stainless Wires

All reagents were purchased commercially and used as received without any further purification. Chemically bonding-type MPC polymer was prepared by the method already reported [PCT/JP2017/024574] (United States Patent No. US 11,015,078 B2 and Japan Patent No. 6964344). Tetraethoxysilane (TEOS) and triethoxymethylsilane (MTES) were added to 1-butanol and fully mixed. Ethanol, phosphoric acid, and distilled water were added to the resulting solution as per the ratio of TEOS and MTES/1-butanol/ethanol/water/phosphoric acid (1/7/7/7/0.05) for preparing the silane coupling coating solution. The stainless steel wire was treated by the oxygen plasma reactor (PR500, Yamato Kagaku, Tokyo, Japan); the O_2_ flow rate was set at 25 mL/min and power at 50 W for 5 min to clean and increase the reactivity of the surface of the stainless wire. Subsequently, the stainless wire was immersed in the silane coupling coating solution for 1 h and dried in the oven at 100 °C overnight. The silane coupling-treated stainless wire was thoroughly washed with distilled water repeatedly and dried. Further, the wire was immersed in the MPC water solution (0.5 wt%) for 1 h and dried in the oven at 100 °C overnight. The MPC polymer-coated stainless wire was washed thoroughly with distilled water and dried.

### 2.2. Effect of MPC-Coated Wires on Frictional Forces

**Figure 1:**
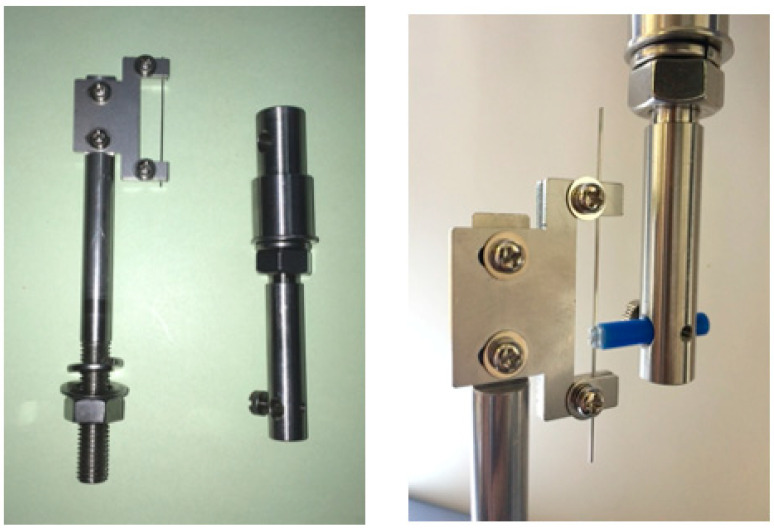
Friction jig and friction test.

This study aimed to investigate the frictional force between the MPC wires using a precision universal/tensile tester AGX (Shimadzu, Kyoto, Japan) and a bracket friction (BF)-exclusive jig (Nishin-Seiki, Hiroshima, Japan; Figure 1). The BF-exclusive jig was attached to AGX and fixed with a screw after adhesion of the bracket (Mini-Clippy 0.018 × 0.025 inch slot, Roth-type, upper canine bracket (Tomy International Inc., Tokyo, Japan)) to the traction division. Thereafter, 0.016 × 0.022 inch stainless steel wires (Tomy International Inc., Tokyo, Japan) were coated with MPC, and the wires were fixed to the wire-grasping part of the BF-exclusive jig. Subsequently, artificial saliva (Saliveht^®^ Aerosol (Teijin Pharma, Tokyo, Japan)) was applied to the wire and bracket at 25 °C (Viscosity of 4–6 mm^2^ s ^−1^) for 1 mL.

The pull-out test was performed at a cross-head velocity of 0.1 mm/s, and the maximal friction coefficients produced by the wires and brackets were measured. The experiment was performed as three repeats of triplicates under the same conditions.

The lower surface of the jig is designed to mount the wire, whereas the top surface of the jig is designed to mount the bracket. The coefficient of friction between the bracket and the wire is measured quantitatively by pulling up the jig on the upper surface at a speed of 0.1 mm/s upward.

### 2.3. Antimicrobial Effects of MPC-Coating Treatment

The MPC coating was added on a 50 × 50 mm stainless steel plate, and the antimicrobial activity of the sample was tested with reference to Japanese Industrial standards (JIS) Z 2801:2012 “Antimicrobial processed products-Antimicrobial test method and antimicrobial efficacy” (JIS 2801:2010 Amendment) [25]. The Japan Food Research Laboratories (Tokyo, Japan) was requested to conduct the antimicrobial test, and the following experimental method was used.

First, 50 × 50 mm test pieces of an MPC-processed stainless metal plate, an unprocessed stainless metal plate, and a glass plate (control group) were placed in sterilized petri dishes, after which 0.2 mL (*S. mutans*: 1.4 × 10^6^ mL; *Porphyromonas gingivalis* (*P. gingivalis*) 1.0 × 10^7^ mL) of a bacterial solution containing the test bacteria (*P. gingivalis* (JCM 8525) or *S. mutans* (IFO 13955)) was added to the center of the sample. To treat the stainless metal plate such that the test organism solution was uniformly seeded, a polyethylene film cut into 40 × 40 mm was placed on top of the test organism solution. The sample was incubated at 35 °C for 24 h.

For *P. gingivalis*, the bacterial suspension was inoculated in 1/5 Gifu anaerobic medium, and anaerobic culture was performed.

Subsequently, the inoculated test organisms were washed out. In other words, each of the coated film and test piece was placed in a separate petri dish while exercising caution not to spill the bacterial suspension. Further, 10 mL of the medium was added to the dish, the test organisms on the unprocessed test specimens were washed out with a scalpel pipette at least four times, and the bacterial fluid was completely recovered. Subsequently, viable bacilli counts per cm^2^ of specimen were determined by the plate culture method.

For *P. gingivalis*, a plate culture method with brucella agar supplemented with 5% equine defibrinated blood was performed, with anaerobic incubation at 35 °C for 5–7 days. After incubation, the number of viable bacilli count per cm^2^ of specimen in the petri dish was determined. For *S. mutans* bacterium, a wash-out solution was applied to soybean-casein-digest broth medium, and the resulting mixture was incubated at 35 °C for 48 h. After incubation, the number of viable bacilli count per cm^2^ of specimen in the petri dish was determined.

The antimicrobial activity values were calculated using the following formula:

R = Ut − At

where R is the antibacterial activity, Ut is the mean logarithm of the viable count (/cm^2^) of the control specimen at 24 h, and At is the mean logarithm of the viable count (/cm^2^) 24 h after the culture. Five experiments were performed for evaluating the antibacterial effect of MPC-coating treatment, and the respective antibacterial values were calculated.

### 2.4. Effects of MPC-Coated Wires on Experimental Tooth Movement In Vitro

To verify the effect of MPC polymer-treated wires on experimental tooth movement models in vitro, we examined the improvement in tooth movement efficiency using typodonts, which are used by orthodontists in clinical training. A typodont articulator (TYPO; YDM Corporation, Tokyo, Japan) was fitted with metal teeth (MTWs; Nisshin Dental Products Inc., Kyoto, Japan) on dedicated normal occlusal waxes, and the maxillary and mandibular first molars on both sides were extracted.

**Figure 2:**
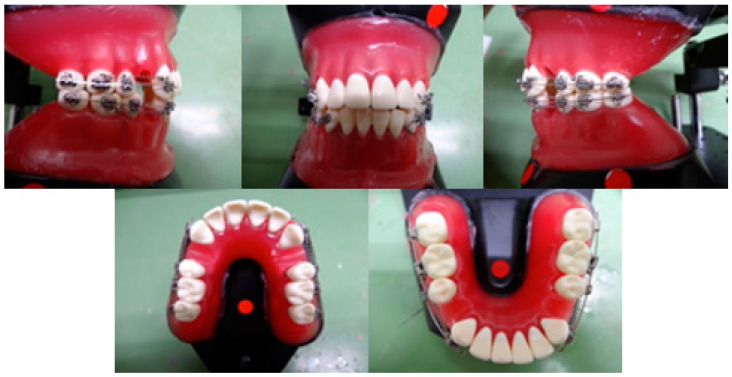
Effects of 2-methacryloyloxyethyl phosphorylcholine (MPC)-coated wires on experimental tooth movement in vitro.

Subsequently, indirect bonding with a bracket (Mini-Clippy Formula R: 0.018 × 0.025-inch slots, Roth type; Tomy International Inc., Tokyo, Japan) was performed. The bracket was attached to the maxillary and mandibular canines, second premolars, first molars, and second molars. They were fitted with a stainless steel wire (right side: non-treated control, left side: MPC-coated wire). The maxilla was subsequently fitted with a 50 g closing coil spring (Sentaroy^®^; Tomy International Inc., Tokyo, Japan) between the bracket hook of the canine and the first molar. Contrarily, the lower jaw was fitted with a 100 g closing coil spring (Sentaroy^®^; Tomy International Inc., Tokyo, Japan) between the bracket hook of the canine and the first molar (Figure 2). Thereafter, the tooth was allowed to penetrate at 50 °C for 20 min, the canine tooth movement pattern was photographed, and measured with a dental caliper. The experiment was performed as three repeats of triplicates under the same conditions.

### 2.5. Statistical Analysis

All data were processed with Bell Curve for Excel software (Society Survey Research Information Co., Ltd., Tokyo, Japan). Descriptive statistics, including standard deviation, mean, and frequency, were used for all variables.

After assessing the normal distribution of data, the Mann–Whitney U test was used for comparisons between groups. *p* values < 0.05 was considered significant.

## 3. Results

### 3.1. Effect of MPC-Coated Wires on Frictional Force

**Figure 3:**
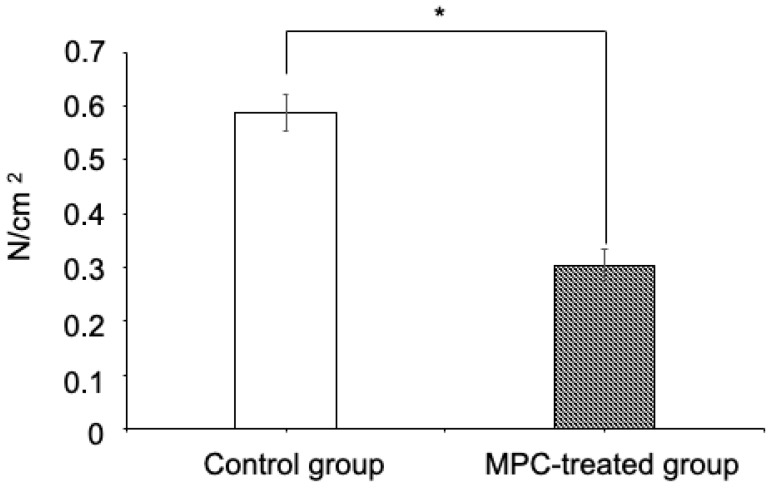
Effect of MPC-coated wires on frictional force. Data are presented as mean ± SEM of three independent experiments (*n* = 3). The Mann–Whitney U test was used for comparisons between groups. *p* value < 0.05 was considered significant. * *p* < 0.05. MPC, 2-methacryloyloxyethyl phosphorylcholine; SEM, standard error of the mean.

The MPC-coating treatment group showed a significantly lower friction coefficient than the non-treatment group (Figure 3).

### 3.2. Antimicrobial Effects of MPC Coating

**Figure 4:**
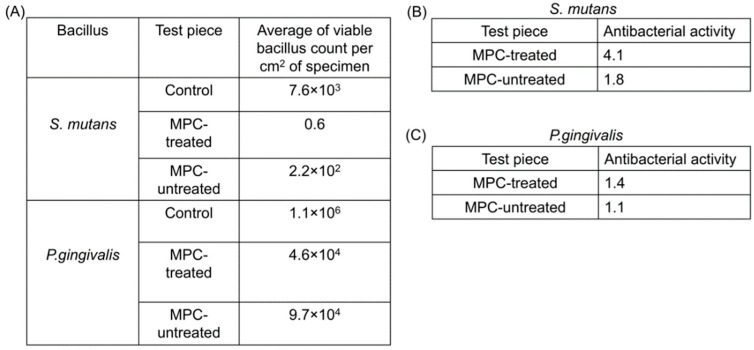
Antimicrobial effects of MPC-coating treatment. (**A**) The results of antimicrobial tests for *Streptococcus mutans* and *Porphyromonas gingivalis*; (**B**) The results of antibacterial activity for *Streptococcus mutans*; (**C**) The results of antibacterial activity for *Porphyromonas gingivalis*.

The result of the antimicrobial tests for *S. mutans* and *P. gingivalis* bacteria on the stainless metal plates are shown in Figure 4A. 

For *S. mutans*, the average viable bacilli count per cm^2^ of the specimen was 7.6 × 10^3^ in the control group and 2.2 × 10^2^ in the group with metal plates without MPC coating. Therefore, the antibacterial activity was 1.8 (Figure 4B). On the other hand, the MPC-coated metal plate group showed a remarkably low value of 0.6, and antibacterial activity showed a high antimicrobial value of 4.1 (Figure 4B). As for *P. gingivalis*, the average viable bacilli count per cm^2^ of the specimen was 1.1 × 10^6^ in the control group and 4.6 × 10^4^ in the non-MPC-coated group; hence, the antibacterial activity was 1.4 (Figure 4C). In contrast, in the MPC-coated group, the average viable bacilli count per cm^2^ of the specimen was 9.7 × 10^4^, and antibacterial activity was 1.1 (Figure 4C). No major changes in antibacterial activity in *P. gingivalis* were observed between the MPC-untreated and MPC-treated groups.

(A) Results of antimicrobial tests for *S. mutans* and *P. gingivalis* bacteria on stainless metal plates.
(B) Results of antimicrobial activity values for *S. mutans.*

Five experiments were performed for evaluating the antibacterial effect of MPC-coating treatment, and the respective antibacterial values were calculated. The antibacterial product was determined to evaluate the antibacterial effectiveness. The optimal antibacterial activity using testing methods of these standards is 2.0. The antimicrobial activity of the MPC-coated group against *S. mutans* was 4.1, while that of the MPC-untreated group was 1.8.

(C) Results of antimicrobial activity values for *P. Gingivalis.*
(D) The antimicrobial activity of the MPC-coated group against *P. Gingivalis* was 1.4, while that of the MPC-untreated group was 1.1.

MPC, 2-methacryloyloxyethyl phosphorylcholine; *S. mutans, Streptococcus mutans; P. gingivalis*, *Porphyromonas gingivalis*.

### 3.3. Effects of MPC-Coated Wires on Simulated Artificial Tooth Movement In Vitro

**Figure 5:**
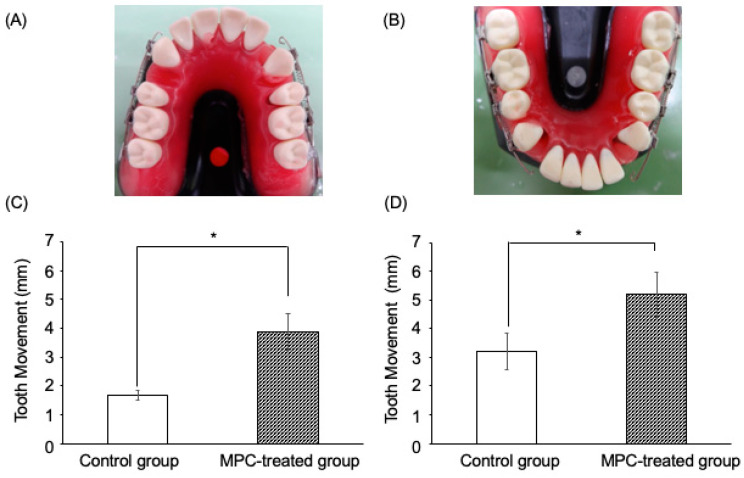
Effects of MPC-coated wires on experimental simulated artificial tooth movement in vitro. (**A**) The maxillary model; (**B**) The mandibular model; (**C**)The results of tooth movement in the maxilla (**D**) The results of tooth movement in the mandibule.

The polymer treatment wire groups had significantly (*p* < 0.05) enhanced simulated artificial tooth movement in experimental tooth movement models compared with the untreated wire groups, at both 50 g and 100 g traction forces (Figure 5C,D).

The results are shown in the images. It was observed that the MPC-coated group had a greater amount of simulated artificial tooth movement than the control group. In addition, the MPC-coated group was observed to have a wire extending into the mesial portion of the canine due to efficient migration (A and B). In the experimental simulated artificial tooth movement model, the MPC-treated group showed greater tooth movement than the control group (C and D).

The polymer-treated group showed significantly (*p* < 0.05) enhanced simulated artificial tooth movement in the experimental tooth movement models compared with the untreated group at both 50 g and 100 g traction forces. The data are represented as mean ± SEM of three independent experiments (*n* = 3). * *p* < 0.05.

MPC, 2-methacryloyloxyethyl phosphorylcholine; SEM, standard error of mean.

## 4. Discussion

Coating of medical devices with MPC has been reported to be highly useful in reducing the number of adherent bacteria [26,27,28]. In addition, the efficacy and safety of MPC polymers as biomaterial are well established and approved by the Food and Drug Administration [28,29,30]. Recently, dental resins were incorporated into MPC to achieve great protein-repellent properties [31]. Moreover, a novel MPC-dimethylaminohexadecyl methacrylate composite has been shown to possess strong and durable resistance to protein adhesion and potent bacteria-eradicating function while matching the load-bearing ability of a commercial dental composite [32]. MPC was incorporated into resin-modified glass ionomer cements, yielding protein-repellent effects, antibacterial properties, and calcium and phosphate ion release [33]. Furthermore, adding the MPC polymer to calcium silicate-based cements has been shown to confer protein-repellent properties and reduce bacterial attachment, with the potential for improved mineralization [34]. In addition, a clinical study reported that 5% MPC polymer mouthwash suppresses total oral bacteria and *Fusobacterium nucleatum* in the oral cavity [35]. These reports indicate that suppression of bacterial adherence in the oral cavity may be expected by applying this polymer to stainless steel wires as primary orthodontic materials. The antimicrobial activity level in this study was 1.8 in the antimicrobial test against *S. mutans*—a caries-associated bacterium—in the MPC non-treated stainless steel plate group. Contrarily, the MPC-coated stainless steel plate group showed a high value with an antibacterial activity value of 4.1. An antimicrobial test for *P. gingivalis*, a bacterium associated with periodontal disease, showed an antimicrobial activity of 1.1 in the MPC non-treated stainless steel plate group. In contrast, the MPC-coated stainless steel plate group showed an antimicrobial activity value of 1.4, which is slightly higher than that of the MPC-non-treated stainless steel group. Horita et al. revealed that MPC polymer application markedly inhibited both the adherence and biofilm formation of *S. mutans* on saliva-coated hydroxyapatite and streptococcal adherence to oral epithelial cells and reduced the adherence of *Fusobacterium nucleatum* to streptococcal biofilms. Additionally, a small-scale clinical trial revealed that mouth-rinsing with MPC polymer inhibited the oral bacterial increase, especially those of *S. mutans* [12]. Yumoto et al. demonstrated that MPC polymer treatment significantly reduced the adherence of periodontal pathogens, such as *P. gingivalis*, to oral epithelial cells, and the subsequent TLR2-mediated innate immune response protected oral epithelial cells from chemical irritants [30]. Moreover, graft polymerization of MPC to acrylic denture base material has been shown to reduce the adhesion of *Candida albicans* [36]. Although the present study results are similar to those described previously, there have been no reports confirming the inhibition of *S. mutans* and *P. gingivalis* growth by immobilizing MPC on stainless steel plates. To our knowledge, this is the first report on the effect of *S. mutans* and *P. gingivalis* growth by immobilizing MPC on stainless steel plates.

The importance of friction in clinical orthodontics has received much attention, mainly because reduced resistance to sliding would reduce the length of time required to align teeth and/or close spaces [37]. In orthodontic tooth movement, the resistance to sliding is divided into three components: (1) friction caused by contact of the wire with the bracket surfaces; (2) binding, which occurs when the tooth tips or the wire flexes so that there is contact between the wire and the corners of the bracket, and (3) notching, which occurs when permanent deformation of the wire occurs at the wire–bracket corner interface. Tooth movement stops when a notched wire catches on the bracket corner and resumes only when the notch is released [37]. The present study revealed that the MPC coating of stainless steel wires significantly reduced the friction coefficient compared with the untreated wire. Moreover, in vitro studies on experimental tooth movement with polymer-treated wires significantly increased tooth movement by approximately two-fold in the experimental tooth movement model compared with untreated wires, both at 50 g and 100 g traction forces. Currently, multi-bracket devices have sizes of 018″ × 025″ and 022″ × 028”. In this study, we used a multi-bracket device of 018” × 022” archwire. The reason for using archwires with a size of 016” × 022” is that archwires with a size of 016” × 022” in a multibracket device of 018” × 025” are the main wires used for tooth movement. However, other archwires, such as 017” × 025” archwire, have not been investigated. Therefore, a similar study will be necessary in the future using an archwire of another size and a multi-bracket device of 022” × 028”.

The MPC treatment significantly reduced the friction between the bracket and the wire, potentially by preventing binding and notching, and thereby allowing efficient experimental tooth movement. These results suggest that application of the MPC polymer on the stainless-steel wires used as a primary orthodontic material may reduce bacterial adherence in the oral cavity and improve tooth movement.

This in vitro study has some limitations.

(1) In the experimental simulated artificial tooth movement model of this study, it was suggested that MPC-coated wires could significantly promote the amount of tooth movement and reduce the friction coefficient to about half. However, the mechanical aspect of tooth movement is complex. Therefore, further analysis and elucidation using FEM analysis is required.
(2) Approximately 800 species of oral bacteria have been reported in an actual oral cavity, each showing complex interactions and forming biofilms. In this study, only a single bacterial species was cultured and evaluated for verification in vitro. Therefore, this is not representative of the actual oral environment. Based on the study results, it is necessary to further investigate the effects of MPC in vivo. In addition, clinical and translational research is needed to verify changes among oral flora of humans using next-generation sequencers.
(3) There are several clinical applications of materials in the medical field. However, MPC-coated materials have not been approved for clinical application in the dental field. Further studies are necessary to confirm the safety on oral tissues and the absence of adverse events.
(4) Tooth/bracket movement in orthodontics shows major interactions involving the teeth, periodontal ligament, alveolar bone, and forces of mastication. Hence, the findings of this in vitro study cannot be used to describe the efficacy and safety of MPC wires. Therefore, further studies, including clinical and translational research, are necessary to elucidate the safety of MPC wires and their effects on the mode of movement in teeth.

## 5. Conclusions

In conclusion, coating stainless steel with MPC led to the suppression of oral bacteria from adhering to the coating and an attenuated friction coefficient, and the hypothesis of achieving smooth experimental tooth movement was supported with data. The findings further suggest that the MPC coating allowed efficient tooth movement by reducing the occurrence of friction. Thus, the application of the MPC coating on stainless steel wires may reduce bacterial adherence in the oral cavity and improve tooth movement.

## Data Availability

The data underlying this article are available on reasonable request from the corresponding author.
